# Validation of preoperative variables and stratification of patients to help predict benefit of cytoreductive nephrectomy in the targeted therapy ERA

**DOI:** 10.1590/S1677-5538.IBJU.2015.0118

**Published:** 2017

**Authors:** Brandon J. Manley, Eric H. Kim, Joel M. Vetter, Aaron M. Potretzke, Seth A. Strope

**Affiliations:** 1 Washington University School of Medicine, Division of Urology, St. Louis, Missouri, USA

**Keywords:** Carcinoma, Renal Cell, Kidney Neoplasms, Nephrectomy, Risk Factors, Comorbidity

## Abstract

**Objectives:**

To further elucidate which patients with metastatic renal cell carcinoma (mRCC) may benefit from cytoreductive nephrectomy (CN) before targeted therapy (TT), and to assess the overall survival of patients undergoing CN and TT versus TT alone.

**Materials and Methods:**

We identified 88 patients who underwent CN at our institution prior to planned TT and 35 patients who received TT without undergoing CN. Preoperative risk factors described in the literature were assessed in our patient population (serum albumin, liver metastasis, symptomatic metastasis, clinical ≥T3 disease, retroperitoneal and supradiaphragmatic lymphadenopathy). Patients were stratified by number of pretreatment risk factors and overall survival (OS) was compared.

**Results:**

TT patients had significantly more risk factors compared to CN patients (3.06 vs. 2.11, p<0.01). Patients who received TT alone had median OS of 5.8 months. All but one patient receiving TT alone had two or more risk factors. A comparison of the CN and TT groups was performed by constructing Kaplan-Meier curves. There was no significant difference in median OS for those patients with exactly two risk factors (447 vs. 389 days, p=0.24), and those with three or more risk factors (184 vs. 155 days, p=0.87).

**Conclusions:**

Using previously described pretreatment risk factors we found that patients with two or more risk factors derived no significant survival advantage from CN in the TT era. These risk factors should be incorporated in the assessment of patients for CN.

## INTRODUCTION

Based on the results of two landmark randomized controlled trials (1-3), cytoreductive nephrectomy (CN) is recommended as a part of the treatment for many patients with metastatic RCC (mRCC). Multiple studies have addressed appropriate patient selection for CN (4-7). Various pretreatment risk factors have been proposed, including serum albumin, serum lactate dehydrogenase (LDH), retroperitoneal or supradiaphragmatic lymphadenopathy, liver metastasis, symptomatic metastasis on presentation, and clinical T3 or greater disease (6). However, validation of these risk factors in an independent data set is lacking, especially among those treated with targeted therapy (TT) alone. Importantly, previous studies included patients who received immunotherapy, rather than TT, which is not consistent with contemporary practice.

To better determine the benefits of CN in the TT era, we retrospectively reviewed patients at our institution who received TT alone (TT group) or CN followed by planned TT (CN group). We assessed the ability of previously identified risk factors (6) to discriminate survival in our population. Patients were stratified by the number of risk factors present, and the overall survival of patients undergoing CN and planned TT was compared to those receiving TT alone.

## MATERIALS AND METHODS

### Patient population

After Institutional Review Board approval, we retrospectively reviewed all mRCC patients who received systemic TT from 2005 to 2013 at our institution. We defined TT as patients who received tyrosine kinase inhibitors (TKI), mammalian target of rapamycin (mTOR) inhibitors, or vascular endothelial growth factor (VEGF) inhibitors. We identified 100 patients who underwent CN at our institution prior to initiation of planned TT, and 39 other patients who received TT without undergoing CN. Of the CN patients, eight were excluded due to unavailable pretreatment imaging and four were excluded due to missing survival data. Of the TT patients, two were excluded due to incomplete clinical data and two were excluded due to prior immunotherapy. Thus, our final cohort consisted of 123 patients: 88 patients who received CN and 35 who were treated with TT alone. Histologic subtype for the CN patients consisted of: 71% clear cell, 17% sarcomatoid, 7% papillary type II, 5% other (including collecting duct, chromophobe, and squamous differentiation). Subtype classification for the TT alone patients could not be determined as all patients were diagnosed based on biopsy of their metastatic site, which presented histologic limitations.

### Clinical variables and outcomes

The following clinical variables were collected: age, adult comorbidity evaluation (ACE) score (8), Karnofsky performance status, serum albumin, serum lactate dehydrogenase (LDH), clinical T stage, presence of liver metastasis, symptomatic metastasis, and retroperitoneal or supradiaphragmatic lymphadenopathy. Clinical T stage was based on the 2010 American Joint Committee on Cancer (AJCC) staging system. AJCC clinical N and M stage were not recorded, as the data points pertaining to lymphadenopathy and metastasis were selected for their previously demonstrated significance (9). Pretreatment albumin was not available for 11 of the 123 patients (8%) included in our study. Pretreatment LDH was not available in 81of 123 patients (65%). Although we chose to remove LDH from our primary analysis, a sensitivity analysis was performed using multiple imputation to ensure no significant changes occurred due to missing data. Pretreatment risk factors used in our primary analysis were: serum albumin below laboratory normal range, clinical T3 or T4 disease, presence of liver metastasis, symptomatic metastasis, and retroperitoneal or supradiaphragmatic lymphadenopathy >1cm. Survival data was gathered using available medical records and the Social Security death index. Our final query of the death index was on October 6, 2013.

### Statistical analysis

#### Baseline patient characteristics

Continuous variables were compared with the paired t-test, and categorical variables were compared with chi-squared testing. Statistical significance was defined by p<0.05 (two-tailed). Median overall survival for CN patients stratified by risk factor group versus TT only.

An attempt was made to validate the findings of Culp et al. in which they ascertained risk of death based on number of risk factors in CN patients (6). Therefore, CN patients were stratified by the number of pretreatment risk factors present. TT patients were not subdivided and considered the referent for this analysis. Univariate Cox proportional hazards analysis was performed for each CN risk factor group and compared to all TT patients. Culp et al. conducted a Cox proportional hazards analysis for CN patients with three or fewer and four or more risk factors. As LDH was removed from our analyses, the model was completed for CN patients with two or fewer risk factors and those with three or more risk factors.

## Survival analysis

Kaplan-Meier estimated overall survival (OS) was compared between CN and TT groups stratified by the number of pretreatment risk factors present. As only one patient with fewer than two risk factors received TT alone without CN, all patients with fewer than two risk factors were excluded from the Kaplan-Meier analysis. There was no comparator group for patients with fewer than two risk factors receiving CN. The remaining patients were stratified into two groups: exactly two risk factors or three to five risk factors. Log rank p-values were calculated to compare survival curves. Multivariate cox proportional hazards analysis was also performed adjusting for age and comorbidity.

## Sensitivity analysis

Multiple imputation analysis was performed for missing data. For each missing variable, multiple imputations were derived at random on the basis of the distribution of each variable within our data. All statistical analyses were then repeated with imputed values for LDH and albumin to ensure no changes resulted from missing data. All statistical analyses were completed using R software, version 2.15.1 using the package ‘survival’ for the survival analysis (10) and the package ‘MICE’ for multivariate imputation and analysis (11).

## RESULTS

Baseline patient clinical characteristics are provided in Table-1. Mean number of risk factors was significantly greater for the TT group as compared to the CN group (3.06±1.08 vs. 2.11±1.17, p<0.01). Significantly more patients in the TT group had symptomatic metastasis (77% vs. 53%, p=0.02) and supradiaphragmatic lymphadenopathy (63% vs. 30%, p<0.01) as compared to the CN group. After CN, 14/88 (15.9%) CN patients did not undergo the previously planned TT; justifications included death (7.50%), refusal (2,14%), no evidence of disease (2, 14%), and decision in consultation with medical oncology to undergo active surveillance (3,21%).

**Table 1:** Baseline patient clinical characteristics.

Variable	CN	TT	p-value
Number of patients	88	35	
Mean age (SD), years	57.4 (10.4)	57.8 (10.4)	0.87
Mean ACE score (SD)	1.22 (0.96)	1.26 (1.01)	0.83
Karnofsky performance ≤ 60 (%)	10/88 (11%)	12/35 (34%)	**<0.01**
Mean number of risk factors (SD)	2.11 (1.17)	3.06 (1.08)	**<0.01**
Albumin ≤ 3.5 (%), g/dL	61/88 (69%)	20/35 (57%)	0.20
Clinical stage ≥ T3 (%)	36/88 (41%)	13/35 (37%)	0.70
Liver metastasis (%)	18/88 (20%)	11/35 (31%)	0.20
Symptomatic metastasis (%)	47/88 (53%)	27/35 (77%)	**0.02**
Retroperitoneal LAD (%)	32/88 (36%)	19/35 (54%)	0.07
Supradiaphragmatic LAD (%)	26/88 (30%)	22/35 (63%)	**<0.01**

**CN =** cytoreductive nephrectomy; **TT =** targeted therapy; **SD =** standard deviation; **ACE =** adult comorbidity evaluation; **LAD =** lymphadenopathy

**Figure 1:**
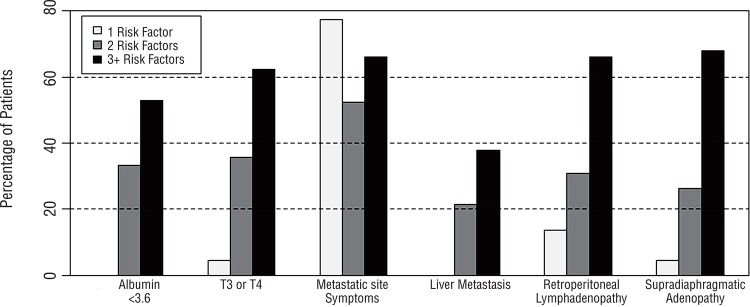
Distribution of individual risk factors among patients stratified by total number of risk factors present.

Figure-1 illustrates the distribution and frequency of individual risk factors stratified by the total number of risk factors present for each patient. For patients with exactly one risk factor, symptomatic metastasis was seen most frequently (17/22=77%). For patients with exactly two risk factors, symptomatic metastasis was also seen most frequently (24/42=57%), but all risk factors were represented. For patients with three or more factors, all risk factors were similarly represented: clinical stage ≥T3 (32/53=60%), symptomatic metastasis (33/53=62%), retroperitoneal lymphadenopathy (33/53=62%), and supradiaphragmatic lymphadenopathy (33/53=62%).

Median OS for CN patients stratified by risk factor group compared to all TT patients are provided in Table-2. TT patients were not stratified and served as the referent group. Patients who received TT alone without CN had a global median OS of 5.8 months. Patients who received CN and had two or fewer factors demonstrated median OS of 22.1 months. In univariate analysis, when compared to all TT patients as a referent, this represented significantly greater OS (HR=0.39, 95% CI 0.23-0.65). OS did not significantly differ between all TT patients and CN patients with three or more risk factors (HR=1.29, 95% CI 0.74-2.23).

**Table 2:** Median overall survival for CN patients stratified by risk factor group versus TT only.

Patient Group	N	HR	95% CI	P-value	Median OS, months
**TT Only**	35	Reference	-	-	5.8
					
**CN**					
**Risk Factors**					
0	6	0.47	0.11-1.99	0.30	15.5
1	21	0.30	0.15-0.63	**<0.01**	28.3
2	30	0.49	0.27-0.88	**0.02**	14.9
3	23	1.08	0.59-1.96	0.80	7.2
4	4	1.31	0.45-3.80	0.62	4.7
5	4	5.64	1.72-18.5	**<0.01**	2.0
< 2	57	0.39	0.23-0.65	**<0.01**	22.1
> 3	31	1.29	0.74-2.23	0.37	6.1

**CN =** cytoreductive nephrectomy; **TT =** targeted therapy; **HR =** hazard ratio; **CI =** confidence interval; **OS =** overall survival

**Figure 2:**
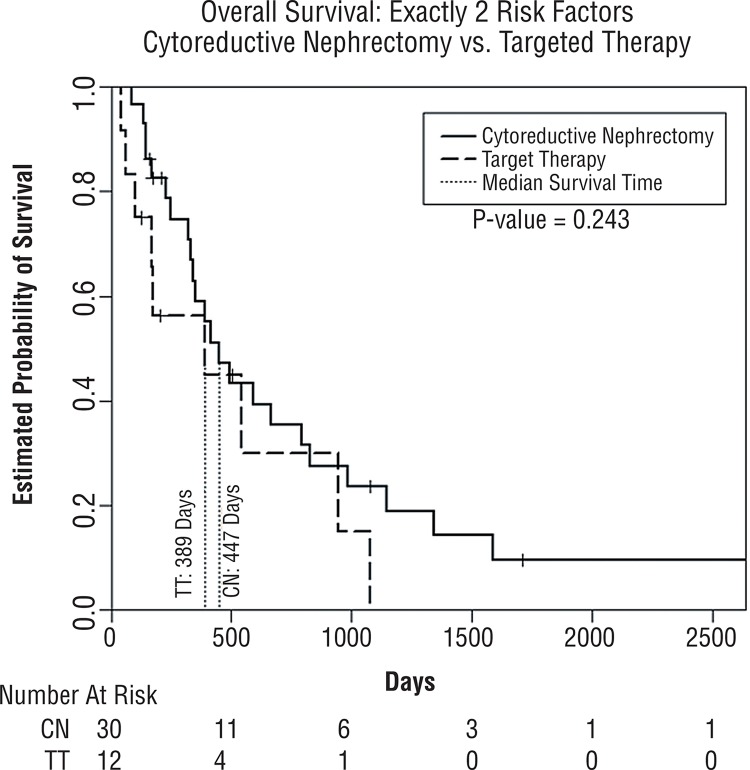
Kaplan-Meier estimated survival comparing patients in cytoreductive nephrectomy group (CN) to targeted therapy group (TT) for patients with exactly two risk factors.

**Figure 3:**
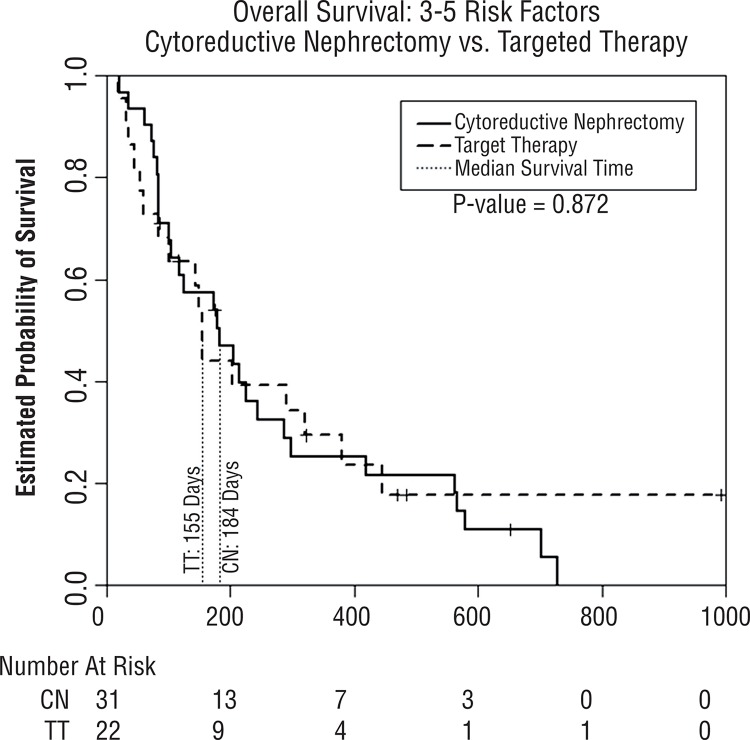
Kaplan-Meier estimated survival comparing patients in cytoreductive nephrectomy group (CN) to targeted therapy group (TT) for patients with three to five risk factors.

Table-3 demonstrates the median OS from our Kaplan-Meier analysis for both the CN and TT groups stratified by their number of risk factors. No patients in the TT group had zero risk factors, and only one had one risk factor. Due to this we were not able to carry out comparative analysis between CN patients with 0-1 risk factors and TT patients with 0-1 risk factors. Stratification was performed to provide CN and TT groups with exactly two and three or more risk factors. Figure-2 illustrates the estimated survival curves for patients with exactly two risk factors. No significant difference in median OS was noted between CN and TT only (447 vs. 389 days, p=0.24) for a difference of about 2 months. Figure-3 illustrates the estimated survival curves for patients with three or more factors. For these patients, no significant difference in median OS was noted between CN and TT only (184 vs. 155 days, p=0.87), for a difference of about 1 month. Furthermore, after stratification by number of risk factors present and multivariate analysis controlling for age and comorbidities, CN did not demonstrate a benefit in either those with exactly two risk factors (HR=1.37, 95% CI 0.56–3.38) or three or more risk factors (HR=0.87, CI 0.47–1.63).

**Table 3:** Kaplan-Meier analysis with median overall survival for both the cytoreductive nephrectomy and targeted therapy groups stratified by number of risk factors.

	Cytoreductive Nephrectomy		Targeted Therapy	

Factors	Count	Median Overall Survival (days)	Count	Median Overall Survival (days)
0	6	466.5	0	*
1	21	850	1	*
2	30	447	12	389
3	23	215	10	156
4	4	142.5	8	232
5	4	60.5	4	152

* = unable to calculate due insufficient data

All analyses were repeated using the imputed values for LDH and albumin levels. The association of increasing numbers of risk factors and worsening survival remained intact in this analysis. CN was not associated with improvement in survival for patients with two or more risk factors in these analyses.

## DISCUSSION

We found that median overall survival for patients undergoing CN decreased as their number of preoperative risk factors increased. When the CN patients were stratified by preoperative risk factors and compared to the TT patient group as a whole, we found an apparent survival advantage for CN in patients with fewer than or equal to two risk factors (Table-2). However, after stratifying both CN and TT patients by the number of preoperative risk factors present, we found no differences in survival when exactly two or three or more pretreatment risk factors were present for each group, based on Kaplan-Meier analysis (Figures 2 and 3). Likewise, multivariate analysis controlling for age and ACE did not demonstrate a benefit of CN for patients with two or more risk factors.

An attempt was made to validate the findings by Culp et al. in their analysis of risk factors in CN patients (Table-2) (6). Like the group from M.D. Anderson, we used the medical therapy cohort as the referent group. The noted difference in the present univariate analysis is that we were unable to include LDH as an assessed risk factor due to missing data in a large portion of our cohort (65%). Also, in our preliminary analysis patients with three risk factors did not derive a benefit from CN. Therefore, rather than assess patients with three or fewer and four or more risk factors as was done by Culp and colleagues, we stratified CN patients by two or fewer and three or more risk factors. Ultimately, our results mirror those of Culp et al. The analysis is challenging given the heterogeneity and inherent bias in the targeted therapy group, wherein nearly every patient had two or more risk factors. To account for these differences, we derived conclusions from Kaplan-Meier survival curves and multivariate Cox proportional hazards models. In agreement with several other studies, we found the actual benefit of CN may be limited to a select patient population (6, 7, 12). In cases where two or more risk factors were present, patients did not appear to benefit from CN in our stratified analysis.

Interestingly, only a single patient with fewer than two risk factors received TT alone, highlighting a selection bias for patients undergoing CN versus receiving primary TT. In practical terms, it appears that urologists and medical oncologists are already selecting patients at highest risk to forgo extirpative therapy. This seems to be largely based on intuition as our cohort largely predates the work by Culp et al. that defined these risk factors (6).

Often, patients are unable to complete the planned medical therapy after CN. In the present cohort, 14/88 (15.9%) CN patients did not undergo the intended TT. In 2010, Kutikov et al. published their series of 141 patients who underwent CN (13). The authors found that 31% of patients did not undergo the intended systemic therapy. In this group, the medical therapy was omitted due to rapid disease progression (30%), decision for surveillance by oncology (21%), patient refusal (23%), and death (19%). Additionally, of the patients who did receive TT after CN, approximately half of the patients (33/69=47%) received a second-line TT due to either progression of disease or medication intolerability, and the decision of what medication to use as a second or third-line therapy was made at the discretion of the treating medical oncologist. Future studies with larger sample sizes may be able to identify patient and tumor factors predictive of the need for second or third-line TT.

Previous population-based studies have demonstrated a benefit for patients treated with CN (14-16). However, these population-based studies are not able to compare CN and TT patients with similar pretreatment risk factors. This inability to accurately assess disease burden between treatment groups is a clear limitation. Furthermore, many previous studies have included patients who received immunotherapy, presented with asynchronous metastasis, and received radiotherapy (6, 14-16). Although our patient population is relatively small, we have removed many of these confounding factors. The present study includes mRCC patients treated only in the TT era, with no treatment contamination with immunotherapy or radiotherapy. Additionally, we stratify not only our CN patients by pretreatment risk factors but also our TT patients as well. With both CN and primary TT patients stratified by number of risk factors, our study provides additional insight as to which patients may derive benefit from CN. For patients with exactly two and three to five risk factors, we found that performing CN prior to TT over TT alone provides minimal or no improvement in survival. While our study is underpowered to prove that this small survival improvement is statistically significant, even in an appropriately powered study this improvement must also be balanced with the known increased risk of surgical complications in mRCC patients (17).

Our study is not without limitations. It is logical to assume based on our data that the perceived overall health and corresponding prognosis of each patient biased the initial treatments offered. Although the healthier patients, as judged by the surgeon, are more likely to receive CN, we found no evidence for improved survival over TT when data were analyzed stratified by the number of risk factors present. As stated previously, the overwhelming majority of patients with zero to one risk factor underwent CN at our institution, which prevents accurate comparisons between CN and primary TT in this lower risk patient population. Although the TT alone group is a heterogenous treatment group (receiving TKI, mTOR inhibitors, and VEGF inhibitors), the patients who were able to receive TT after CN were expected to have similar heterogeneity in their TT treatments. The choice of specific TT agents to use and when to withdraw or change therapy was made at the discretion of the treating medical oncologist and patient, and was not defined by an institutional or study protocol. While 15.9% of patients in the CN group did not complete the intended TT, this limitation is common in the literature (13). Furthermore, the histologic subclassification in the TT alone group could not be determined as biopsies were performed on metastatic sites and limited in tissue. Future studies with larger sample sizes should examine the histologic subclassification of mRCC and identify if a strong association between histology and survival exists even when diagnosed at a metastatic stage. Finally, our results are from a tertiary care center, and may not reflect the full spectrum of metastatic RCC patients seen in community practice.

Paramount to the practicing urologic surgeon is the relative weight of the risks and benefits of CN. In a population-based series of 16,285 patients by Trinh et al., the overall complication rate was 31% (17). Moreover, the complication rate was increased in those with numerous comorbidities and more than one metastatic site. Additionally, the in hospital mortality rate was 5%, and was significantly greater in those with age ≥75 (7.9%), three or more comorbidities (7.7%), and two or more metastatic sites (7.4%). While the possible benefit of CN is enticing, the results presented herein show it may be prudent to forgo surgery in those with advanced disease or with significant medical illness. Avoiding costly complications and untoward patient suffering is of vital concern.

## CONCLUSIONS

Using previously described preoperative risk factors we found that patients with two or more risk factors derived no significant survival advantage from CN in the TT era. These risk factors should be incorporated in the assessment of patients for CN.
